# Microfluidic Electrospray Microcapsules With Spatiotemporal Release of Asiatic Acid and Baicalein Nanocarriers for Wound Healing

**DOI:** 10.1002/smmd.70047

**Published:** 2026-07-13

**Authors:** Zhiqiang Luo, Lingbao Zeng, Xinyu Zhu, Yuanjin Zhao

**Affiliations:** ^1^ School of Biological Science and Medical Engineering Southeast University Nanjing China; ^2^ Department of Rheumatology and Immunology Nanjing Drum Tower Hospital School of Pharmacy, Nanjing University of Chinese Medicine Nanjing China

**Keywords:** asiatic acid, baicalein, herb, liposome, microfluidics, wound healing

## Abstract

Synergy therapy of Chinese herbs is an effective strategy for wound repair and its further development focuses on the chronological release of specific herbs with healing proceeding. Herein, we report a novel asiatic acid/baicalein nanocarrier integrated microcapsule with spatiotemporal release feature from microfluidic electrospray for wound healing. Benefiting from advantages of both nanocarrier formulation and integration capacity of microfluidic electrospray, synthesized baicalein‐tannic acid nanoparticles are integrated into the shell of microcapsule while fabricated asiatic acid liposomes are located at the core region. This design enables chronological release: baicalein is first released to kill bacteria, followed by releasing asiatic acid liposomes to enhance cell migration and granulation tissue formation. In vitro tests confirm the excellent biocompatibility, antibacterial and pro‐migration property of microcapsules. The results from in vivo wound healing studies showed the outcomes of reduced inflammation and accelerated wound closure in microcapsules‐treated wounds. Therefore, it is believed that this herbal microcapsule with spatiotemporal and hierarchical release of baicalein and asiatic acid is an effective therapeutic platform for clinical wound treatment.

## Highlights

- AA liposomes were derived from concentrated extrusion of AA and phospholipid molecule.
- Baicalein‐tannic acid nanoparticles (BTANPs) were synthesized by solvent exchange.
- AA liposomes and BTANPs were integrated into ABNM by microfluidic electrospray.
- ABNM could chronologically release BTANPs and AA liposomes with their biodegradation.
- ABNM were highly curative to the healing of wounds.

## Graphical abstract

Asiatic acid (AA) liposomes are derived from concentrated extrusion of AA and phospholipid molecule. Baicalein‐tannic acid nanoparticles (BTANPs) are synthesized by solvent exchange. They are integrated into the different regions of hydrogel microcapsules by microfluidic electrospray, obtaining AA/BTANPs integrated microcapsules. They can chronologically release BTANPs and AA liposomes with the biodegradation of hydrogel to highly promote wound healing.

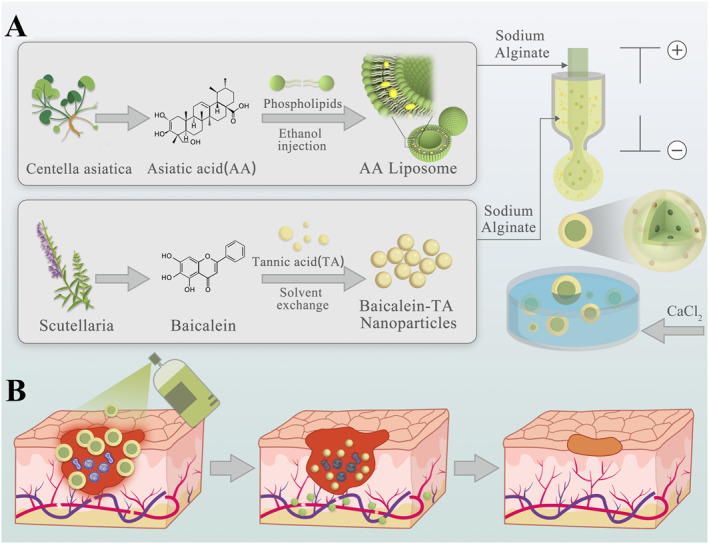

## 1 Introduction

Wound repairing is a complicated and highly programmed process, generally known as four overlapped phases: hemostasis, inflammation, proliferation and remodeling [1, 2, 3, 4]. Over the thousands of years of medication practice, massive traditional Chinese herbs have been reported to be highly therapeutic for wound healing [5, 6, 7]. Various herbs that meet the different demands of healing stages are combined in clinical administration to struggle for best healing outcomes. For example, baicalein, an active ingredient of scutellaria, has good antibacterial properties to reduce the risk of bacterial infection in the early stages of trauma [8, 9, 10, 11]. Asiatic acid (AA), the active ingredient of *Centella asiatica*, is normally applied in the late stages to improve collagen synthesis [12, 13, 14]. Especially, with the advances in delivery systems in recent years, these herbs are further loaded into hydrogels to enhance the delivery efficiency and therapeutic effects [15, 16]. Despite achieving some developments, they still face several difficulties. Generally, the existed hydrogel block is not fit to the irregular wound and is not flexible enough for convenient application. Additionally, the release of the drugs loaded within the hydrogel lacks release controllability, which may hamper the healing process of the wound. Therefore, new herb‐based therapeutic strategies with spatiotemporal release profiles are still anticipated.

**FIGURE 1:**
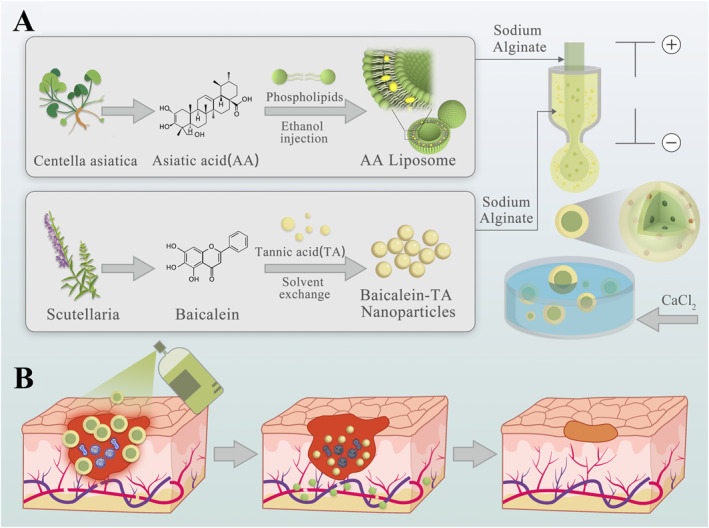
Illustration of ABNM fabrication and curative effects for promoting wound healing. (A) Illustration of ABNM fabrication. (B) Therapeutic effects of ABNM on infected wounds.

Herein, we proposed a novel AA/baicalein nanocarriers integrated microcapsules (ABNM) with spatiotemporal release feature from microfluidic electrospray for wound healing (Figure 1). Microfluidics electrospray that is a technique for manipulating liquids at the micron scale under an applied electric field, has been utilized to prepare functional microcapsules with precisely manipulated shapes and ingredients [17, 18, 19]. It facilitates the manufacture of drug delivery systems with incorporation of multiple drugs and controllable release profile [20]. In contrast, nanocarrier formulation is a promising approach to overcome the drawbacks of poor solubility, low delivery dosage and bioavailability of traditional Chinese herbs. Among various nanocarriers, liposomes and complex nanoparticles are two of the most advantageous forms. Liposomes, composed of phospholipid bilayers, can encapsulate lipophilic drugs to ease their delivery and prevent potential mutual interactions between other drugs [21, 22, 23, 24]. In addition, complex nanoparticles at the nanoscale dimension possess unique advantages in the enhancement of delivery dosage and bioavailability [25, 26, 27, 28]. Therefore, it is conceived that utilizing microfluidics to integrate nanosized herb derivatives may construct a new delivery system of poorly soluble herbs.

In this work, we fabricated the desired microfluidic microcapsules with nanosized herb derivatives for wound healing. AA liposomes were derived from concentrated extrusion of AA and phospholipid molecule while baicalein‐tannic acid nanoparticles (BTANPs) were synthesized by solvent exchange. Microfluidic electrospray technology was used to integrate AA liposomes into the core region and BTANPs into the shell layer, respectively. The resultant ABNM could chronologically release BTANPs and AA liposomes with the biodegradation of the calcium‐alginate (Ca‐Alg). The shell layer preferentially degraded to release baicalein, which can prevent bacterial infection. After total degradation of the shell and initial degradation of the core region, AA liposomes within the capsule were sustainably released to promote cell migration. Cell tests demonstrated the excellent biocompatibility, antibacterial and pro‐migration properties of ABNM. Additionally, ABNM demonstrated a superior ability to inhibit inflammatory responses, promote granulation tissue formation and collagen deposition, which significantly accelerated wound healing in *in vivo* animal tests. These findings indicated that the proposed microcapsules with spatiotemporal and hierarchical release of herb derivatives hold promise for improving wound healing.

## 2 Results and Discussion

**FIGURE 2:**
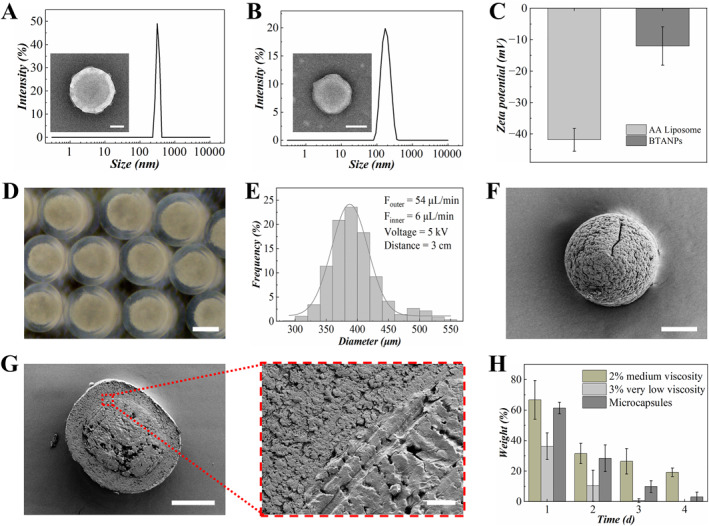
Characterization of ABNM. (A) The TEM image of BTANPs and its dynamic light scattering (DLS) results. (B) TEM image of AA liposome and its DLS results. (C) Zeta potential of AA liposome and BTANPs. (D) Optical microscopy image of ABNM. (E) The size distribution of ABNM. (F, G) The images of (F) a microcapsule, (G) cross‐section of a microcapsule and its interface between the shell and core, observed under SEM. Scale bars represent 100 nm in (A, B); 200 μm in (D), 100 μm in (F, G) and 10 μm in enlarged image. (H) The degradation data of microcapsules along with time.

In a typical study, baicalein was first dissolved in dimethyl sulfoxide (DMSO) and then rapidly mixed with tannic acid aqueous solution under continuous stirring. In this process, baicalein and tannic acid molecules formed BTANPs via hydrogen bonding. Compared to baicalein molecule that is almost insoluble in water, the fabricated BTANP suspension was stable colloidal solution state (Supporting Information S1: Figure S1A,B). They presented a spherical morphology, as observed by transmission electron microscopy (TEM) (Figure 2A). Their average hydrodynamic particle size was 321.06 nm while their zeta potential was −12.03 mV (Figure 2A,C). AA liposomes were derived from concentrated extrusion of AA and phospholipid molecules. Specifically, AA was dissolved together with cholesterol and phosphatidylcholine in ethanol solution, and then the mixture was rapidly injected into phosphate buffer saline (PBS) under stirring for hydration. After evaporation of ethanol, the mixture was repeatedly injected in an extruder with a polycarbonate membrane with 200 nm pores. Given the fact that AA is hydrophobic, AA was loaded into the gap of the lipid bilayer, obtaining the uniform AA liposomes (Supporting Information S1: Figure S1C). They were multi‐lamellar vesicles with distinct fingerprint‐like morphology, as observed under TEM (Figure 2B). The measured hydrodynamic particle size of the AA liposomes was about 181 nm and their zeta potential was approximately −41 mV (Figure 2B,C). These two nano‐sized herb derivatives had a relatively higher drug loading capacity than their free molecule forms, making them more suitable for sustained release.

Microfluidics method was employed to integrate BTANPs and AA liposomes into the specific region of ABNM, achieving administration of their delivery efficiency. A microfluidic device was constructed by coaxial assembly of two capillaries (Supporting Information S1: Figure S2). The inner capillary was at about 140 μm of inner diameter and the outer capillary had an inner diameter of about 320 μm. During the laminar flow of fluids in the microfluidic channels, the fluid of 2% sodium alginate (Na‐Alg, medium viscosity) suspended with BTANPs in the outer phase encapsulated the inner fluid of 3% Na‐Alg (very low viscosity) suspended with AA liposomes. Droplets were then generated at the nozzle of the microfluidic device under the applied voltage and sprayed into a 2% calcium chloride (CaCl_2_) solution. Na‐Alg precursor was solidified into Ca‐Alg hydrogel once contacted with CaCl_2_ solution, forming ABNM with a core–shell structure, as shown in Figure 2D. The overall diameter of ABNM was 397.02 ± 41.33 μm in a typical fabrication process, which was pretty uniform (Figure 2E). It is worth noting that the diameter of the microcapsules was tunable with the change in the collection distance, voltage, and flow rates of outer phase (F_outer_) and inner phase (F_inner_). When other factors were fixed, the diameter of ABNM was increased with the increase of the F_inner_ and F_outer_. When the collection distance was decreased or the applied voltage was increased, the size of the microcapsules decreased (Supporting Information S1: Figure S3). Furthermore, the detailed structure of the ABNM was specifically explored using scanning electron microscopy (SEM). The surface of ABNM had a rough and dense texture in both the surface and interior (Figure 2F,G). The boundary between the core and shell interface of the microcapsules was clear, presenting a distinct layered structure that was conducive to hierarchical release of drugs.

The drug release profile of herbs nanocarriers from ABNM is a crucial property for drug delivery, which was along with the biodegradation of Ca‐Alg hydrogel. Therefore, this study evaluated the degradation performance of ABNM and the release capacity of nanosized herb derivatives from ABNM. In the degradation test, homogeneous microspheres from 2% medium viscosity Na‐Alg and 3% very low viscosity Na‐Alg were also fabricated to investigate the influence of material difference on the degradation rate of ABNM. The collected data demonstrated that microspheres from 2% medium viscosity Na‐Alg hydrogel degraded slower than that from 3% very low viscosity Na‐Alg. The ABNM presented a similar rate to microspheres of 2% medium viscosity Alg at the initial process and 3% very low viscosity Alg at the later process (Figure 2H). After that, the release profiles of BTANPs located in the shell layer and AA liposomes located in the interior region from ABNM were quantified in PBS and incubated at 37°C. The results demonstrated that both the BTANPs and AA liposomes were gently released from the ABNM, performing sustained release property. During the initial release period (0–6 h), the release rate of the BTANPs was significantly higher than that of the AA liposomes. After approximately 24 h, the release of the BTANPs entered a plateau phase (with the cumulative release rate approaching 80%). Meanwhile, the release rate of the AA liposomes was very slow before 6 h. Then, it began to obviously accelerate and sustainably released after 36 h (Supporting Information S1: Figure S4). This differential release behavior could be attributed to the core–shell structure of the ABNM: the shell layer was directly exposed to the medium and preferentially degraded to release the BTANPs while the core layer was encapsulated by the shell layer to delay the drug release. All the above data demonstrated the excellent drug‐carrying and hierarchical release capabilities of ABNM.

**FIGURE 3:**
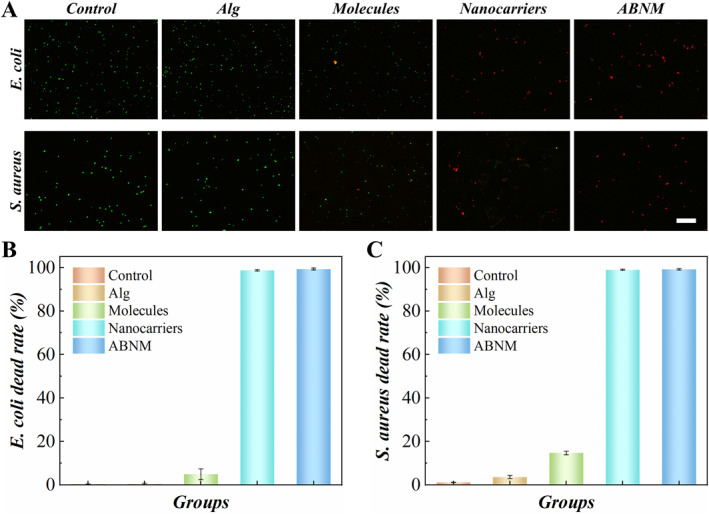
In vitro antibacterial testing of ABNM. (A) Live/dead staining results of bacteria in five groups. (B, C) Dead rates of (B) *E. coli* and (C) *S. aureus*. Scale bar is 100 μm in (A).

Wounds are inevitably exposed to the surrounding environment, leading to bacterial infections. Baicalein could damage membranes by decreasing the amount of lipids, upregulating cell wall hydrolysis‐related genes and increasing the antibiotic sensitivity. To evaluate the antibacterial effects of ABNM, *Escherichia coli* (*E*. *coli*) and *Staphylococcus aureus* (*S*. *aureus*) were selected as model bacteria. There were five groups, including Control group (no extra treatment), Alg group (incubated with blank microcapsules with no herbs), Molecules group (incubated with two herb molecules), Nanocarriers group (incubated with two nano‐sized herbs derivatives) and ABNM group (incubated with ABNM). As expected, nano‐sized herb derivatives and ABNM greatly killed both the *E*. *coli* and *S*. *aureus*. In contrast, the dead ratio of bacteria in the Molecules group was much lower than the nanocarriers‐related groups. This was mainly attributed to the poor water solubility of baicalein and AA, making them difficult to effectively act upon bacteria (Figure 3). These findings demonstrated the high antibacterial efficiency of nano‐sized herb derivatives. Additionally, their encapsulation into the shell of ABNM did not obviously decrease their antibacterial performance.

**FIGURE 4:**
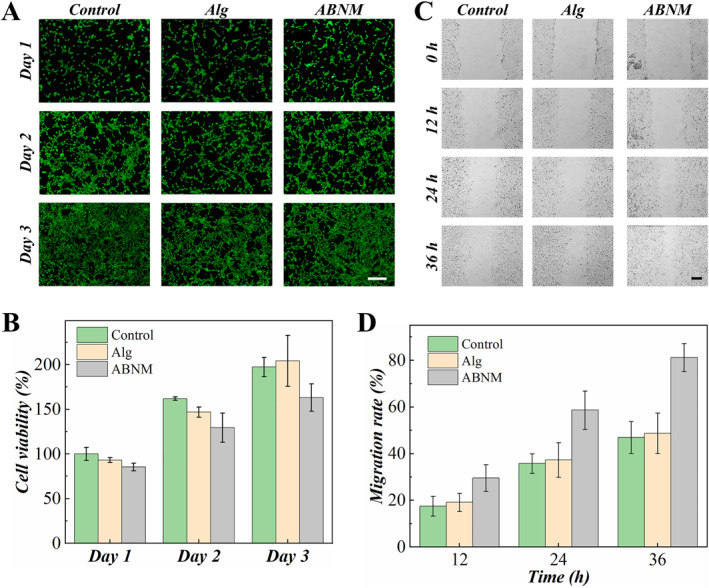
Biocompatibility and cell scratch test of ABNM. (A) Fluorescence images of NIH‐3T3 cells over 3 days. (B) Cell viability measured by MTT assay. (C) Migration images of HUVECs received different treatments at 12th, 24th, and 36th h. (D) Statistical analysis of migration rates of cells in different groups. Scale bars represent 200 μm in (A) and (C).

Biocompatibility of the delivery system is crucial to avoid potential toxic effects. To assess the biocompatibility of ABNM, NIH‐3T3 cells were co‐cultured in complete medium with no extra additive as Control group, with the addition of extraction medium from blank Alg microcapsules as Alg group, and with extraction medium from ABNM as ABNM group, respectively. The fluorescence images of cells stained by Calcein‐AM and measured cell viability via MTT assay demonstrated the rapid proliferation and consistently healthy morphology of 3T3 cells in all the groups along 3 days, validating the high biocompatibility of the fabricated ABNM (Figure 4A,B). Subsequently, to further validate the pro‐migration effect of ABNM that was derived from AA liposomes, a scratch test was conducted. Human umbilical vein endothelial cells (HUVECs) monolayer after scratching was treated with incomplete medium with no extra additive (Control group), extraction medium of blank Alg microcapsules (Alg group), and extraction medium of ABNM (ABNM group), respectively, for testing. As illustrated in Figure 4C,D, the ABNM group exhibited the highest migration rates at all the time points. After 36 h, its migration rate exceeded 80%, significantly outperforming both Control group and Alg group. This outcome confirmed that the AA liposomes could be released from ABNM to substantially enhance cell migration, which was crucial for the formation of new blood vessels and the remodeling of tissues.

**FIGURE 5:**
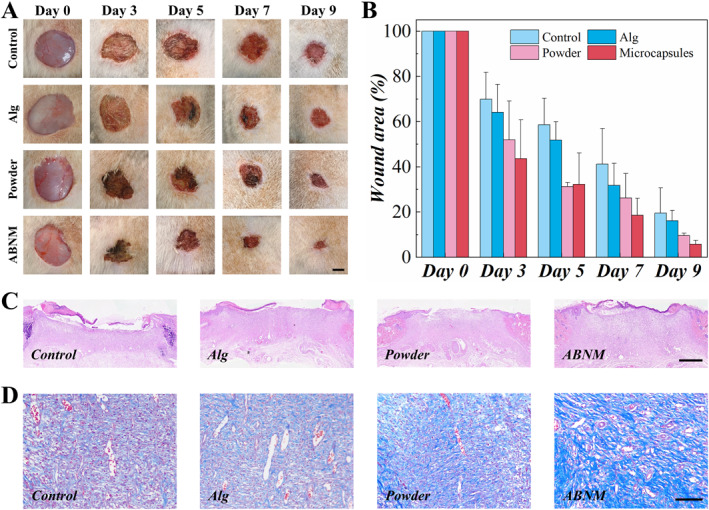
Efficiency of ABNM for accelerating wound closure. (A) Digital photos of the skin wounds that received different treatments. (B) Measured average wound area. (C) Representative images of tissues after H&E staining. (D) Representative images of tissues after Masson's trichrome staining. Scale bars are 0.5 cm in (A), 1 mm in (C) and 100 μm in (D).

Encouraged by the in vitro results, the practical curative efficacy of the ABNM was verified in *in vivo* infected skin wounds on the back of Sprague–Dawley (SD) rats. After wound creation and bacterial infection, these rats were divided into four groups to receive corresponding interventions, including ABNM group (treated with ABNM), Powder group (treated with powder of two herbs), Alg group (treated with blank Alg microcapsules) and Control group (cleaned by PBS). Their healing processes were recorded on day 0, 3, 5, 7 and 9 for further detailed analysis. Wound area was measured to calculate the wound repair rate. As shown in Figure 5A,B, the final repair rate of rats in the ABNM group was the fastest on day 9, demonstrating the enhanced drug delivery efficacy, followed by the Powder group, Alg group and Control group in turn. Hematoxylin and eosin (H&E) staining was utilized to observe the thickness of the regenerated tissues on day 9. As shown in Figure 5C and Supporting Information S1: Figure S5A, the data revealed that the thickness of the granulation tissues in all the three groups received therapeutic interventions were thicker than the Control group. The regenerated granulation tissues in the ABNM group showed the thickest tissues, reaching 1.86 ± 0.13 mm, followed by the Powder group (1.62 ± 0.09 mm) and Alg group (1.36 ± 0.09 mm) in turn. These results suggested that herb nanocarriers had a positive effect on the enhancement of the drug delivery efficacy.

Tissue remodeling is a crucial index of wound healing, which could be revealed by the collagen deposition at the wound site. To evaluate the collagen deposition status, Masson's trichrome staining was carried out and the collagen deposition rate was calculated. As shown in Figure 5D, blue collagen after staining deposited the lowest at the tissues in the Control group while that in the ABNM group showed the best level of collagen deposition (Supporting Information S1: Figure S5B). The Powder group also showed more collagen deposition compared to the Control group, but was lower than the ABNM group. The better regeneration of granulation tissues and collagen deposition of the ABNM group indicated their excellent prospects and potential in promoting granulation tissue regeneration for wound healing.

**FIGURE 6:**
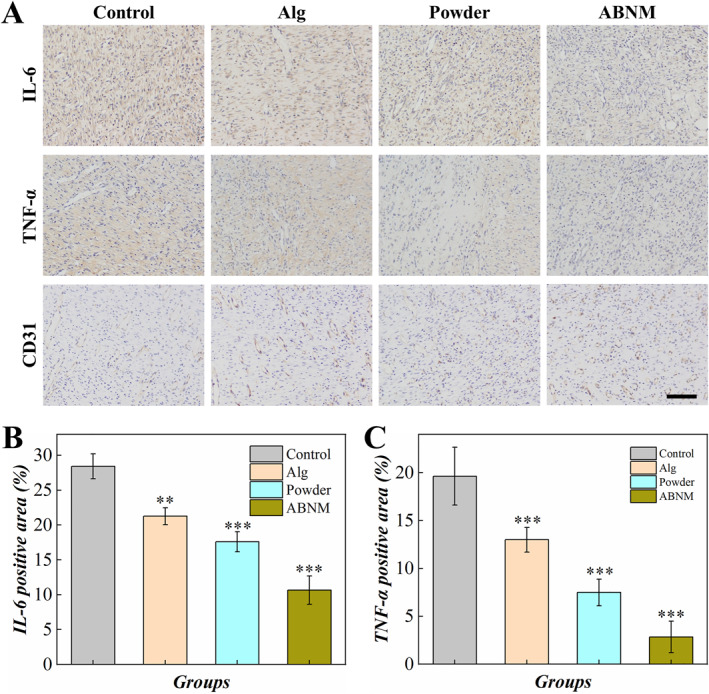
Histological analysis of wound tissues. (A) Immunohistochemical staining results of IL‐6, TNF‐α, and CD31 in wound tissues from different groups on day 9. (B, C) Statistic of (B) IL‐6 and (C) TNF‐α in four groups. Scale bar is 100 μm in (A). **p < 0.01, ***p < 0.001.

Inflammatory response is an essential activity during wound healing. When the wound is infected by bacteria, it is prone to trigger a severe inflammatory response, which directly affects the healing of the wound. Therefore, on day 9 post‐treatment, we detected the secretion level of two typical inflammatory factors, tumor necrosis factor‐α (TNF‐α) and interleukin‐6 (IL‐6). Immunohistochemical staining results revealed that the expression of TNF‐α and IL‐6 in the ABNM group and Powder group presented alleviated inflammation response (Figure 6A). Notably, compared to the Powder group, the ABNM group showed significantly reduced expression of IL‐6 and TNF‐α, decreased to 10.6% and 2.8%, respectively (Figure 6B,C). In contrast, the Control group exhibited severe inflammatory responses. These data demonstrated that the herb nano‐carriers exerted better therapeutic effects and the microcapsule system enabled continuous release of herb nanocarriers to improve wound repair. Angiogenesis is another important indicator of tissue repair status. We performed immunohistochemical staining of platelet endothelial cell adhesion molecule‐1 (CD31) to reflect the neovascularization level at the wound. As illustrated in Figure 6A and Supporting Information S1: Figure S5C, compared to the Alg group and Powder group, the ABNM group demonstrated significantly higher blood vessel density. This enhancement of revascularization in wounds was benefited from the synergistic effects of BTANPs and AA liposomes. In contrast, lower vascular density was observed in the Powder group (powder of both herb compounds). In short, the ABNM with modified herb nano‐carriers substantially boosted the therapeutics of herbs themselves. This multifunctional delivery system demonstrated substantial promise for accelerating infected wound healing.

## 3 Conclusion

In conclusion, we fabricated the Chinese herb microcapsules with two kinds of traditional Chinese herbs, AA and baicalein, for promoting wound healing. BTANPs were synthesized via solvent exchange while AA liposomes were prepared by the co‐extrusion method. BTANPs and AA liposomes facilitated the delivery convenience and enhanced the dosage of their molecules. Subsequently, core/shell Ca‐Alg microcapsules with spatiotemporal release profiles were fabricated by using microfluidic technology. BATNPs and AA liposomes were encapsulated into the shell layer and core layer, respectively. This strategy not only preserved the bioactivity of the herbal components but also minimized the side effects associated with complex chemical processing. Furthermore, based on the synergistic effects of AA and baicalein, these herbal microcapsules demonstrated exceptional performance in antibacterial activity, inflammation relief, collagen deposition, angiogenesis, and tissue remodeling, presenting an ideal therapeutic effect on infected skin wounds. It is believed that ABNM with herb nanocarriers had a good potential in promoting wound healing, providing a new direction for the development and application of traditional poorly soluble Chinese herb ingredients. Additionally, this work provided a new proposal for the construction of a delivery system with a spatiotemporal release profile that was decided by the differential degradation rates and spatial distribution of the hydrogel matrix in the core and shell regions. Through the replacement of hydrogel matrix and drugs, it is possible to tailor the drugs release rate and dosage to achieve more precise, intelligent, and personalized therapeutics according to the disease demand in the fields of tissue engineering, tumor combination therapy, manufacturing of cascaded biological microreactors, etc.

## 4 Experimental Section

### 4.1 Materials

Medium viscosity Na‐Alg was bought from Sigma. Very low viscosity Na‐Alg (Alfa Aesar), CaCl_2_ (Alfa Aesar), cholesterol and soybean phospholipids were bought from Macklin. Baicalein, tannic acid, and AA were bought from Aladdin. Calcein‐AM was supplied by Beyotime. TNF‐*α*, IL‐6 and CD31 antibodies for immunohistochemical staining were provided by ServiceBio. 8–12 weeks SD rats were provided by Qinglong Mountain Animal Breeding Farm.

### 4.2 BTANPs Preparation

25 mg baicalein was dissolved in 1 mL DMSO. 900 mg tannic acid was dissolved in 3 mL deionized water. 261 μL of baicalein‐DMSO solution was dropwise added into 3 mL of tannic acid aqueous solution to get the 2 mg/mL baicalein solution, stirred overnight and kept away from light. After that, the mixture was transferred into a 14 kDa dialysis tube for a 5‐day dialysis treatment, obtaining purified BTANP nanocarriers.

### 4.3 Preparation of AA Liposomes

Dissolve 240 mg soybean phospholipids and 40 mg cholesterol in 2 mL absolute ethyl alcohol, and add 1 mL 700 μM AA‐ethanol solution. Transfer the dissolved lipid solution into a syringe and slowly inject into 10 mL of PBS under rapidly stirring. After 1 h, the resulting mixture was repeatedly injected 20 times in an extruder with a polycarbonate membrane with 200 nm pores. The resultant liposomes were purified in 14 kDa dialysis tube and then stored at 4°C.

### 4.4 ABNM Fabrication

Based on the low‐viscosity of the used Na‐Alg solution, filter with pore sizes of 0.22 μm was used for sterilization of Na‐Alg solution and CaCl_2_ solutions. A filter with pore sizes of 0.45 μm was used for sterilization of BTANPs and AA liposomes suspension. The microfluidic device was consisted of two glass capillaries, coaxially fixed on a glass slide. It was irradiated with ultraviolet light to achieve sterilization. The outer phase fluid was a 2% Na‐Alg solution (medium viscosity) suspended with 1 mg/mL BTANPs. 6% Na‐Alg solution (very low viscosity) was mixed with obtained AA liposomes suspension at the ratio of 1:1, acting as the inner phase fluid. High‐voltage DC power was used to create the electric field. 2% CaCl_2_ solution was placed under a microfluidic electrospray device to collect and solidify the collected droplets. All these fabrication procedures were carried out in a sterile environment.

### 4.5 Characterization

The morphology of BTANPs and AA liposomes was observed under TEM (JEOL, Japan) after 1% phosphotungstic acid staining. Hydrodynamic particle size and zeta potential of BTANPs and AA liposomes were measured by using Zetasizer (Malvern). Surface and internal structures of the ABNM were observed by SEM (Zeiss Ultra Plus, Germany). The macroscopic structure and diameter were assessed using optical microscopy (OLYMPUS BX51). Microplate reader was used to detect the absorbance of samples.

### 4.6 Degradation Test

The homogeneous microspheres from 1 mL 2% Alg (medium viscosity) and 3% Alg (very low viscosity) precursor, respectively, were fabricated as comparison. The same dosage of ABNM was used for testing. They were incubated in 5 mL PBS at 37°C. These microparticles were washed and freeze‐dried once a day for weighting to calculate the residual rate of weight.

### 4.7 Drug Release Study

ABNM containing BTANPs within the shell layer and AA liposomes modified by RhB within the core layer were separately prepared and incubated in 5 mL PBS at 37°C. At the scheduled time point, 200 μL supernate was sucked out as sample, following adding 200 μL of new PBS. The absorbance of the sample with BTANPs was detected at 372 nm to calculate the concentration of BTANPs based on a standard curve. The absorbance of the sample with AA liposomes modified by RhB was detected at 570 nm to calculate the concentration of AA liposomes based on a standard curve. The total release number of nanocarriers was calculated from their concentration.

### 4.8 Anti‐Bacterial Study

Bacteria suspension was centrifuged and resuspended in PBS, and their concentration was adjusted to 0.5 MCF. The obtained bacterial suspension was incubated with blank Alg microcapsules (Alg group), baicalin and AA molecules (Molecules group), BTANPs and AA liposomes (Nanocarriers group), ABNM (ABNM group), and a control group without other treatment. After incubation at 37°C for 24 h, live/dead staining of bacteria was performed by Syto9/PI staining for observation.

### 4.9 Biocompatibility Test

1 × 10^4^ cells were cultured in 48‐well plates and co‐incubated with microcapsule extraction medium (24‐h) for 3 days. At 24, 48, and 72 h, the old culture medium was replaced by 450 μL new culture medium and 50 μL of MTT for incubation at 37°C. After another 4 h, the old medium was replaced by 500 μL DMSO to dissolve the formazan crystals. After complete dissolution of formazan crystals, 100 μL of the solution was sucked out for absorbance detection in a 96‐well plate at 490 nm. Cell viability was represented as the percentage relative to the control group on day 1.

### 4.10 Cell Scratch Test

1 × 10^5^ HUVECs were seeded in 12‐well plates, placed in an incubator (37°C, 5% CO_2_). Once the cells in the plate had grown almost without gaps, discard the medium and scratch with a 200 μL tip. Cells were rinsed three times with PBS to remove unattached cells. Subsequently, 1 mL of incomplete medium with no additive (Control group), extraction medium of blank Alg microcapsule (Alg group), and extraction medium of ABNM (ABNM group) were added to each well. The images of gaps caused by scratching were obtained under a microscope at 12th, 24th, and 36th h.

### 4.11 In Vivo Infected Wound Healing

SD rats underwent excision of full‐thickness skin at a diameter of about 1.5 cm on the back. Wounds were infected with *S*. *aureus* suspension (0.5 MCF) to establish an infected wound model. The wounds received treatments of PBS (Control group), blank Alg microcapsules (Alg group), powder of 2 mg baicalin and 0.5 mg AA (Powder group), and ABNM (ABNM group). On day 3, 5, 7, and nine post‐treatments, wounds were photographed, and wound areas were quantified using ImageJ (*n* = 6). On the ninth day, the rats were euthanized to collect regenerated wound samples. For histopathological and immunohistochemical analyses, tissue samples were fixed for paraffin embedding. 5 μm tissue sections were sliced for Masson staining, H&E staining and immunohistochemical staining of TNF‐α, IL‐6 and CD 31.

### 4.12 Statistical Analysis

The statistical data are presented as mean ± standard deviation. Significance analysis used Student's t‐test to analyze the difference significance between the control group and one experimental group. The difference is significant only when *p* < 0.05.

## Author Contributions

Y.Z. conceived the idea and designed the experiment. Z.L. and L.Z. conducted experiments, data analysis, and initial writing. X.Z. and Y.Z. revised the manuscript.

## Ethics Statement

All animals were carefully handled in accordance with the Laboratory Animal Care and Use Guidelines. The Southeast University Animal Investigation Ethics Committee examined and approved the requests in this experiment (approval number: 20250115003).

## Conflicts of Interest

The authors declare no conflicts of interest.

## Supporting information

Supporting Information S1

## Data Availability

The data that support the findings of this study are available from the corresponding author upon reasonable request.
