# Supplementary material for: Microfluidic Electrospray Microcapsules With Spatiotemporal Release of Asiatic Acid and Baicalein Nanocarriers for Wound Healing
Source: Smart Med. 2026 Jul 13;5(3):e70047. doi: 10.1002/smmd.70047 (PMC13387336; doi:10.1002/smmd.70047)
Supplement: Supplementary file 1 — Supporting Information S1 [file SMMD-5-e70047-s001.docx]

Supporting Information

Microfluidic electrospray microcapsules with spatiotemporal release of asiatic acid and baicalein nanocarriers for wound healing

Zhiqiang Luo, Lingbao Zeng, Xinyu Zhu, Yuanjin Zhao*


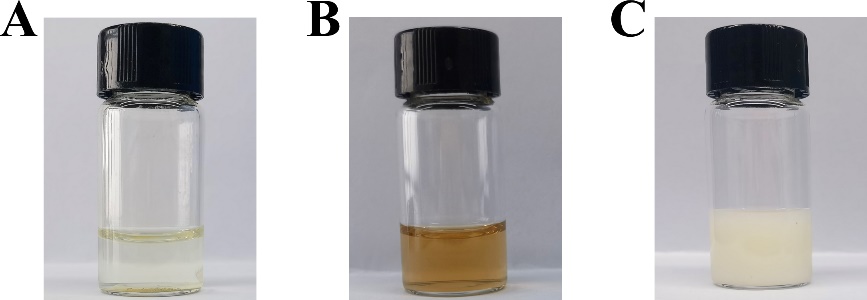


**Figure S1.** Optical images of (A) baicalein, (B) BTANPs suspension and (C) AA liposomes suspension.


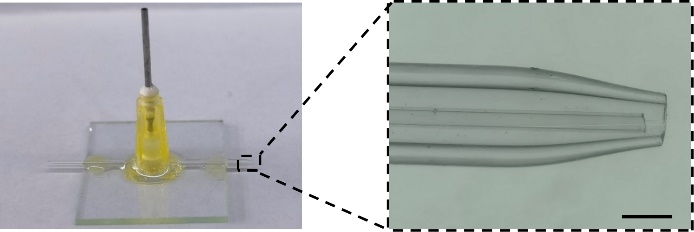


**Figure S2.** Optical images of core/shell microfluidic device. Scale bar is 500 μm.


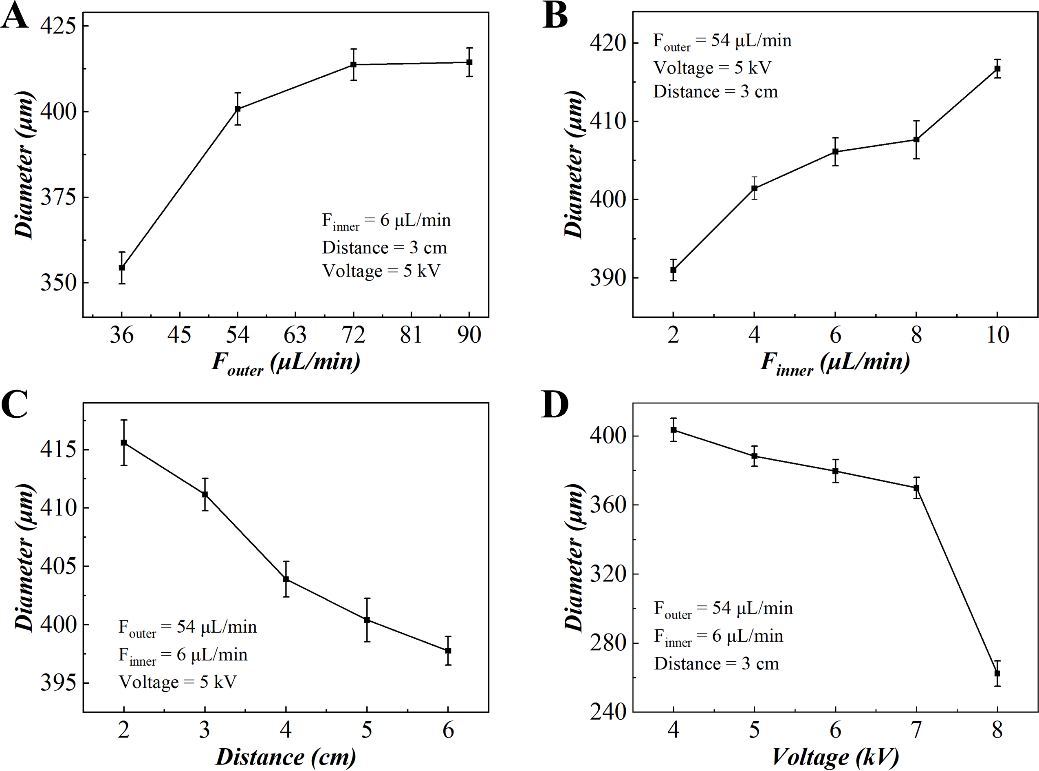


**Figure S3.** Relationships between the microcapsules diameters and (A) outer phase flow rate, (B) inner phase flow rate, (C) collection distance, and (D) applied voltage.


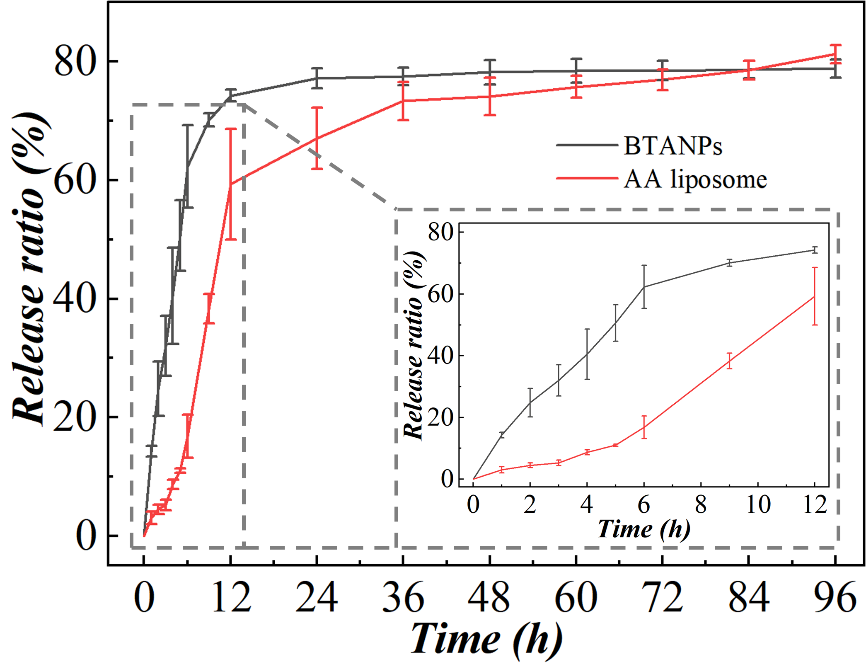


**Figure S4.** Release curve of nanocarriers from ABNM.


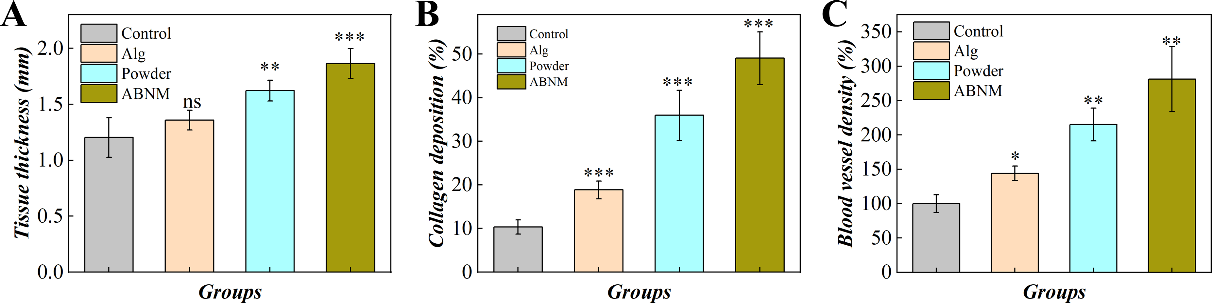


**Figure S5.** Quantitative analysis of staining results. (A) Tissue thickness in different groups on day 9. (B) Collagen deposition area in different groups on day 9. (C) Blood vessel density in different groups on day 9. **p* < 0.05,***p* < 0.01, ****p* < 0.001, ns: not significant.
